# Cell-free microcompartmentalised transcription–translation for the prototyping of synthetic communication networks

**DOI:** 10.1016/j.copbio.2018.10.006

**Published:** 2019-08

**Authors:** Emilien Dubuc, Pascal A Pieters, Ardjan J van der Linden, Jan CM van Hest, Wilhelm TS Huck, Tom FA de Greef

**Affiliations:** 1 Laboratory of Chemical Biology, Department of Biomedical Engineering, Eindhoven University of Technology, 5600 MB Eindhoven, The Netherlands; 2 Institute for Complex Molecular Systems, Department of Biomedical Engineering, Eindhoven University of Technology, 5600 MB Eindhoven, The Netherlands; 3 Computational Biology Group, Department of Biomedical Engineering, Eindhoven University of Technology, 5600 MB Eindhoven, The Netherlands; 4 Department of Biomedical Engineering & Department of Chemical Engineering and Chemistry, Eindhoven University of Technology, P.O. Box 513, 5600 MB Eindhoven, The Netherlands; 5 Department of Physical Organic Chemistry, Institute for Molecules and Materials, Radboud University, Nijmegen 6525 HP, The Netherlands; 6 Institute for Molecules and Materials, Radboud University, Heyendaalseweg 135, 6525 AJ Nijmegen, The Netherlands

## Abstract

Recent efforts in synthetic biology have shown the possibility of engineering distributed functions in populations of living cells, which requires the development of highly orthogonal, genetically encoded communication pathways. Cell-free transcription-translation (TXTL) reactions encapsulated in microcompartments enable prototyping of molecular communication channels and their integration into engineered genetic circuits by mimicking critical cell features, such as gene expression, cell size, and cell individuality within a community. In this review, we discuss the uses of cell-free transcription–translation reactions for the development of synthetic genetic circuits, with a special focus on the use of microcompartments supporting this reaction. We highlight several studies where molecular communication between non-living microcompartments and living cells have been successfully engineered.

## Highlights

• Cell-free transcription and translation systems are used for testing and facilitating the implementation of novel circuits in cells.
• Cell-free transcription and translation systems can be encapsulated in microcompartments.
• Microcompartments are used to study gene expression in a physicochemical environment and at a scale close to those of the cell.
• Microcompartments are capable of communication with cells and other artificial cells and can be used to prototype novel communication channels.

## Graphical abstract

**Figure:**
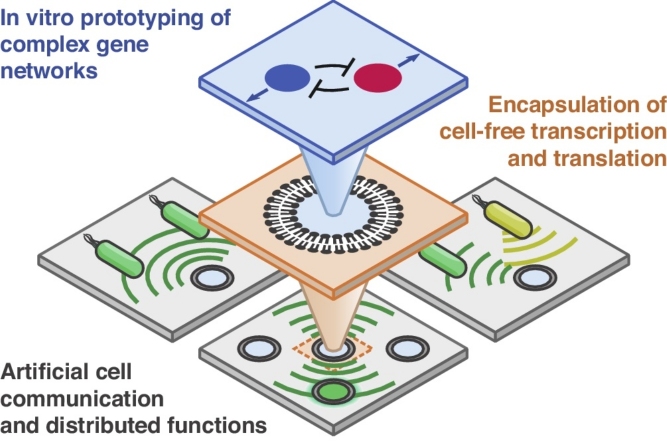

**Current Opinion in Biotechnology** 2019, **58**:72–80

This review comes from a themed issue on **Nanobiotechnology**

Edited by **Giovanni Maglia** and **Wesley R Browne**

For a complete overview see the Issue and the Editorial

Available online 26th December 2018

**https://doi.org/10.1016/j.copbio.2018.10.006**

0958-1669/© 2018 The Authors. Published by Elsevier Ltd. This is an open access article under the CC BY license (http://creativecommons.org/licenses/by/4.0/).

## Introduction

Synthetic biology traditionally utilises genetic circuits in order to implement novel functions in living cells [1,2]. The fundamental approach of synthetic biology is based on the use of building blocks assembled in functional modules, culminating in genetic circuits that can display complex spatiotemporal behaviour such as oscillations, synchronisation and combinatorial control of endogenous signalling pathways [3, 4, 5,6^••^]. However, progressing from module-based to system-focussed synthetic biology remains an ongoing challenge, partly due to our limited knowledge of complex function integration into living systems. For instance, to facilitate the engineering of synthetic cellular consortia capable of displaying collective behaviour, a broader understanding of the mechanisms by which cells communicate in their natural environment is required.

Simplified models of biological systems could help identify key molecular parameters and as such have the potential to uncover generalisable concepts. Proteins and other components of the transcription and translation machinery can be extracted from cells as a lysate or purified and reconstituted (PURE) in order to perform gene expression *in vitro* with a good control over the biochemical composition. Here, compartmentalised cell-free transcription–translation (TXTL) reactions constitute ideal model systems for deducing the rules of network composition, developing new communication pathways between cellular mimics and living cells [7], and creating genetic devices for the implementation of synthetic communication between cells of different species [8].

In this review, we intend to first summarise the use of TXTL reactions for prototyping and implementation of novel genetic networks. We will discuss how microfluidic technologies and semipermeable microcapsules provide essential tools for cell-free synthetic biology in a context where size, composition, and individuality of the reaction are of particular importance. Finally, we will highlight important contributions of encapsulated TXTL to the development of synthetic communication paths.

## Cell-free reactions for applied synthetic biology: circuit testing, behaviour prediction and forward engineering

**Figure 1:**
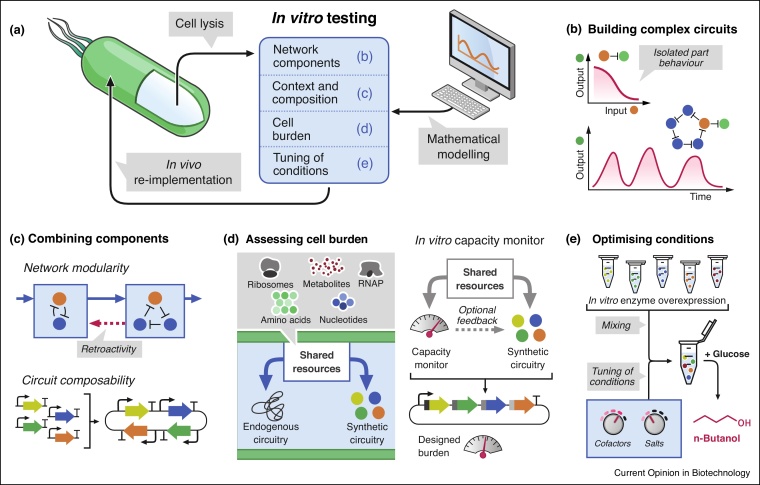
Cell-free reactions for characterising, testing, and optimising complex circuits. Cell-free TXTL reactions can be used in combination with mathematical modelling to test complex circuits, and identify optimal conditions for the implementation of novel biological functions *in vivo***(a)**. Cell-free reactions are used to characterise the behaviour of novel isolated circuit parts [19^••^] (upper panel **(b)**) and test the behaviour of circuits combining novel parts (lower panel (b)). Various versions of similar networks can rapidly be tested in TXTL, which can be used to reveal retroactive interactions between modules (upper panel **(c)**), as well as unexpected effects when combining components into a single construct (lower panel (c)). Cell burden occurs when a synthetic circuit excessively mobilises resources that are also necessary for endogenous circuits (left panel **(d)**). A capacity monitor reports possible cell burden during the implementation of novel circuits using TXTL and allows the design of networks generating minimal burden [21] (right panel (d)). TXTL allows the testing of various biochemical conditions, such as cofactor, salt, and enzyme concentrations, in order to optimise a TXTL-based reaction [30] **(e)**.

As the complexity of synthetic gene networks increases, more resources are shared between synthetic circuits and their host. As a result, cells experience a decrease in fitness, which limits their growth and the efficiency of synthetic circuits [9^••^]. Furthermore, implementing synthetic circuits within cellular hosts exposes these networks to interactions with endogenous pathways, negatively influencing the behaviour of both host and synthetic circuitry. Cell-free synthetic biology has emerged as a powerful tool for testing new genetic networks in a controlled biochemical context, as well as for predicting cellular responses to such networks [10^•^,11^••^]. Here we summarise most recent studies using cell-free TXTL for prototyping novel genetic modules and circuits and facilitating their integration into cellular hosts (Figure 1a).

### Characterisation of novel regulatory parts

Development of complex genetic circuits requires the use of well-characterised regulatory modules. Cell-free TXTL allows the rapid and thorough testing of new genetic parts thus enabling such characterisation (Figure 1b upper panel). DNA and RNA sequence-specific regulation is receiving particular interest as it yields novel regulators with high degree of orthogonality. Researchers recently used a cell-free synthetic biology approach for the testing of novel regulatory elements with further *in vivo* applications, leveraging the strong potential of riboregulation [12^•^,13^•^,^14^,^15^], and dCas9-based repression [16].

### Testing and forward engineering of synthetic gene networks with complex behaviour

After characterising regulatory building blocks, novel genetic circuits can be designed and assembled (Figure 1b lower panel). *In vitro* characterisation is often done under batch conditions, but these conditions do not ensure a constant supply of substrate, as well as the removal of by-products, and the renewal of information, which are prerequisites for the implementation of higher-order regulatory behaviours. In contrast, flow reactors enable the testing of regulatory modules and the implementation of complex networks in conditions mimicking cellular homeostasis [17^••^,18^••^], as well as the forward engineering of such networks into bacterial hosts [19^••^]. In addition, TXTL in flow reactors constitutes an effective method for approaching synthetic biology from control theory perspectives, where compositional context, cell heterogeneity and division become controlled parameters instead of poorly defined variables [20]. Finally, we anticipate flow reactors could serve as platform for studying retroactivity (Figure 1c upper panel) between modules or networks sharing common resources, and test different compositions of a network (Figure 1c lower panel).

### Resource burden

Synthetic gene networks mobilise resources essential to the cell, which are often found in limited supply (Figure 1d left panel). In order to rapidly estimate the resource cost of integrating novel synthetic circuits, Borkowski *et al*. coupled a TXTL batch system to a capacitor circuit, which facilitated circuit transposition into cells [21] (Figure 1d right panel). Coupled to a regulatory dCas9-based negative feedback, this circuit could provide direct supervision over the resource consumption of synthetic circuits [22]. By leveraging the possibility to tune parameters critical to the TXTL reaction, such as DNA instructions and DNA concentration, multiple other systems have also been used to screen and identify resource competition [19^••^,17^••^,^23^].

### Tuneability, context, and composition

One major advantage of cell-free TXTL reactions is the possibility to adjust the parameters influencing the performance of a synthetic circuit, whereas *in vivo* methods rely predominantly on host resources and characteristics. Egbert and Klavins established that, in living cells, the performance of a non-endogenous genetic circuit is highly context-dependent, as a network can yield its designed function in one host strain but fails to achieve this function in another strain [24]. The choice of a bacterial host could be guided by identifying *in vitro* which parameters are essential for the desired behaviour and selecting a strain matching these context requirements. To further optimise host context, many regulatory elements critical for protein synthesis can be added or expressed *in situ*, such as MazF ribonuclease [25], GamS [26^••^] and Chi6 [27] DNase inhibitors, GreA/B transcription elongation factors [28], and ClpXP protease [29]. Karim *et al*. composed a mixture of lysates enriched in specific enzymes or with enzymes expressed *in situ* in order to identify major directions for improving the yield of *n*-butanol synthesis [30] (Figure 1e). Furthermore, Yeung *et al*. leveraged the tuneability of TXTL systems in order to show that supercoiling is one of the main factors explaining differences in expression levels within an intergenic context [31].

### Alternatives to *Escherichia coli*

The majority of cell-free synthetic biology work is based on cell extracts from the *E. coli* bacterium. However, other prokaryotes have been used to provide novel TXTL systems, and could become new model organisms due to their interesting features, such as growth traits and facilitated genetic manipulation [23,32, 33, 34, 35]. Furthermore, additional eukaryotic TXTL systems were recently developed, opening methods for *in vitro* studies with a closer focus on human-centred applications [36, 37, 38].

## Engineered compartments to study physicochemical properties of living systems

Cells display a high intrinsic concentration of biomolecules, a reduced volume, and an amphiphilic interface between their internal volume and external environment. Engineering synthetic compartments of controlled size and composition for the conduction of TXTL reaction enables the study of gene expression at the relevant scale. We distinguish three experimental microcompartments that have been employed to perform TXTL or prototype synthetic genetic networks based on cell-free TXTL, first, PDMS-based compartments, second, lipid-based compartments, and third, coacervates, also known as complex aqueous two-phase systems (ATPS).

### PDMS-based microcompartments

#### Complex network implementation

**Figure 2:**
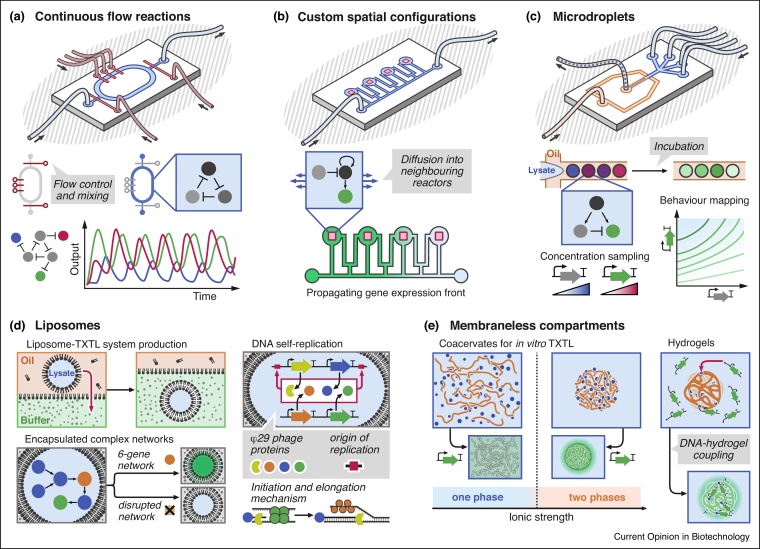
Microfluidic technologies for studying gene expression at the cellular scale. Continuous flow reactors allow the implementation of out-of-equilibrium gene networks in TXTL. Valves precisely control addition and mixing of fresh TXTL reagents, enabling the implementation of complex networks such as oscillators [19^••^] **(a)**. Control over geometry and diffusion allows the study of gene expression propagation and pattern formation in compartmentalised flow reactors using DNA brushes [41] **(b)**. Microdroplets generated on a microchip allow the screening of a vast range of conditions influencing network behaviour in TXTL, such as DNA template concentrations, shown here for the implementation of an incoherent feedforward loop [44] **(c)**. Liposomes encapsulating TXTL can be obtained by double-emulsion techniques (**(d)**, top-left panel). Liposomes are used for isolating genetic cascades [75] ((d), bottom-left panel) and creating units containing self-replicating genetic information [53^••^] ((d), right panel). Upon increase of ionic strength, TXTL mixture can phase separate, resulting in a highly active microcompartment [47] (**(e)** left panel). DNA-functionalised hydrogels are used to create membraneless compartments capable of gene expression ((e) right panel).

In contrast with methods using free-floating DNA gene templates, Bar-Ziv *et al.* proposed a method for performing TXTL reaction on a chip in which the gene template DNA was attached to a functionalised surface, first under batch conditions using wheat-germ extract [39], and subsequently in microfluidic flow reactors using *E. coli* cell extract [18^••^]. The latter, alongside the work of Niederholtmeyer *et al*. [17^••^] (Figure 2a), was amongst the first examples of long-term TXTL, and demonstrated the implementation of advanced synthetic gene networks such as oscillators. Although challenging to fabricate and use, the devices developed by Niederholtmeyer *et al*. are a major technological breakthrough that will facilitate the development of numerous synthetic gene circuits in the future.

#### Reaction-diffusion gene networks

Gene expression-pattern formation plays an essential role in morphogenesis and is a relevant study-case for cell-free synthetic biology. In 2005, Isalan *et al*. implemented a coarse mimic of a *Drosophila melanogaster* morphogenic gene network [40]. Three genes were coupled to paramagnetic beads at defined locations inside a cm-long batch chamber containing wheat germ lysate-based TXTL, yielding the emergence of a reaction-diffusion network allowing the formation of gene expression patterns. This work highlighted important criteria for the implementation of pattern-forming gene networks, first, control over the sublocation of network components, second, control over resource competition and depletion, and third, control over protein degradation. The previously described microfluidic system developed by the Bar-Ziv group provided a platform within which each of these critical criteria can be controlled (Figure 2b). Tayar *et al*. leveraged this technical improvement to study the emergence of expression patterns from out-of-equilibrium gene networks, first using a bistable switch [41], and later a series of oscillators coupled in space [42^••^]. In a follow-up study, Pardatscher *et al*. developed a lithography technique in order to functionalise a surface with up to three distinct DNA strands on a chip supporting both lysate-based and PURE-based TXTL reaction [43]. This technique will allow the study of complex interactions between multiple, spatially resolved, genes and the formation of more complex spatial patterns.

### Lipid-based microcompartments

The cell membrane constitutes an amphiphilic interface between the interior of the cell and its environment. This interface can be mimicked by encapsulating TXTL reactions inside single (water-in-oil) or double (water-in-oil-in-water) emulsions in order to study large number of these reactions at a cellular scale.

#### High-throughput screening

Large numbers of TXTL microdroplets in oil with a controlled dispersity are easy to generate, store, and remain stable over hours, which makes them particularly interesting for screening large numbers of parameters influencing TXTL reactions (Figure 2c). This feature was elegantly employed by Hori *et al*., who optimised a cell-free genetic circuit based on an incoherent feedforward topology using fluorescently barcoded droplets in which lysate-based TXTL reactions can take place [44].

#### Confinement

The reduced size of microdroplets is particularly interesting to study physical effects such as confinement. Guan *et al*. encapsulated Xenopus egg extract in microdroplets of various size. The authors showed that the extract could undergo several mitotic oscillations, and described the influence of the size of the compartments on the period [45]. Sakamoto *et al*. investigated the influence of surface-to-volume ratio of microcompartments on the efficiency of lysate-based TXTL, suggesting a deleterious interaction between membrane lipids and translation machinery [46].

#### Molecular crowding

Macromolecular crowding effects, which emerge in highly concentrated media such as the cytosol, can be artificially induced in TXTL mixtures using crowding additives inside microdroplets. Molecular crowding has been shown to influence the spatial segregation of biomolecules and kinetics of cell-free TXTL reactions [47,48]. In addition, Norred *et al*. showed that noise in transcription reaction in systems displaying reduced diffusion resulted in gene expression bursts, which are likely to occur in cells but are not observable in bulk PURE-based TXTL reactions [49].

#### Semi-permeable liposomes

Given that droplets are intrinsically closed systems, they cannot be utilised for long-term protein expression, nor as analogues of semi-permeable lipid bilayers for mimicking the cell membrane. Noireaux *et al*. first implemented lysate-based TXTL reaction in a liposome permeated by haemolysin pores, which enabled the exchange of nutrients and by-products with a feeding solution, resulting in long-term gene expression [50]. Later, Garamella *et al*. tested various transcriptional cascades inside liposomes [26^••^] (Figure 2d left panel). Majumder *et al*. implemented a lysate-based TXTL liposome sensitive to osmotic changes via expression of the MscL calcium channel [51]. Moreover, lysate encapsulating liposomes were recently used by Krinsky *et al*. to serve as containers for the production and intratumoural delivery of a toxin protein [52]. Finally, Van Nies *et al*. recently described the first liposomes capable of isothermally replicating DNA using self-encoded proteins in PURE system, constituting a major step towards the construction of a true synthetic cell capable of full replication [53^••^] (Figure 2d right panel).

### Membraneless compartments

Under critical ionic concentrations, biomolecules displaying a high electrical charge or high multivalency can undergo complex liquid–liquid phase separation, which yields their partition into membraneless compartments known as coacervates (Figure 2e left panel). Coacervates obtained from TXTL mixture are therefore highly enriched in TXTL machinery. There is growing evidence that phase separation plays a major role in the regulation of protein activity, spatial segregation of nucleic acids, and functional organisation of the cell [54, 55, 56]. Phase separation also gives rise to the emergence of partitioning, confinement, and crowding effects influencing cell-free gene expression [47,57,58]. TXTL was first combined with hydrogels by Park *et al*., who used a micropad of DNA hydrogel incubated in TXTL mixture [59]. The DNA hydrogel, consisting of genes coding for a reporter protein, was not permeable to TXTL machinery, so protein expression occurred at the surface of the gel but not inside. Thiele *et al*. used hyaluronic acid gel beads in which DNA template was covalently attached, and incubated the gel beads in presence of TXTL mixture inside a microdroplet [60]. In contrast to the gel used by Park *et al*., gel beads used in this study were porous enough to allow TXTL to occur inside the gel beads. The authors showed that transcription and translation reactions were confined inside the gel as the mRNA remained trapped inside the beads. Finally Zhou *et al*. produced hydrogel particles containing DNA template, ribosomes, and His-tagged TXTL proteins from PURE system. All biomolecules remained trapped inside the hydrogel particles, so TXTL could occur over several days by continuous supply of feeding buffer (Figure 2d left panel) [61].

## TXTL and communication

Interactions between gut microbiota and the human body recently gained in interest as studies highlighted the impact of microorganisms on the metabolism [62], immune responses [63], and the recurrence of cancer [64]. The development of new biomolecular tools inspired by natural quorum sensing systems is therefore of particular relevance in order to understand multicellular communication [8] and to engineer synthetic communication devices [6^••^,^65^]. Furthermore, to avoid the difficultly of engineering organisms with increasingly complex genetic circuits, the development of orthogonal communication channels that enable distributed functions within a bacterial community has recently received particular attention [8,66]. Nevertheless, the prototyping of synthetic and highly orthogonal molecular communication channels that mediate collective behaviour in populations of living cells remains challenging. Compartmentalised TXTL systems can play an important role in the development of such pathways, as they can mimic communication between cells whilst remaining modular and easy to control.

### Quorum sensor characterisation

Quorum sensors are naturally occurring genetic communication systems found in many bacterial species in a variety of chemical messenger-transcription factor-operator systems. Quorum sensors are essential building blocks for engineering molecular communication channels in synthetic biology [6^••^,^8^]. Halleran and Murray recently characterised a series of quorum sensors using lysate-based TXTL, prior to their implementation *in vivo*, enabling them to accurately predict crosstalk between different systems [67]. In addition, Wen *et al*. used lysate-based TXTL reactions to identify the presence of quorum sensing signals in patients suffering from lung infection by *Pseudomonas aeruginosa*, demonstrating the therapeutic relevance of quorum sensing detection and characterisation [68].

### Artificial cell communication

**Figure 3:**
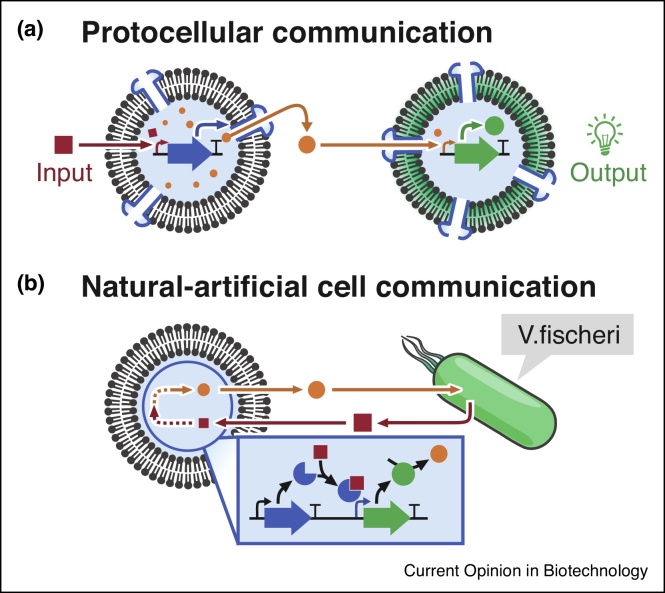
TXTL in liposomes for prototyping novel communication channels. Through the exchange of chemical signalling molecules, liposomes containing TXTL mixture and quorum sensing circuits can be used as artificial cells to establish communication with other artificial cells **(a)** or with living cells **(b)**.

Combination of microfluidic technologies with TXTL allows the generation of cell mimics capable of communication processes based on gene instructions. Booth *et al*. engineered a tissue mimic by 3D-printing TXTL-containing droplets into organised layers [69^•^]. The PURE-based TXTL reactions produced α-haemolysin upon light-activation in order to create communication channels between individual droplets in a programmable manner. In a related approach, Findlay *et al*. programmed specific communication between two droplets by implementing the active transport of a signalling molecule through LacY transporter proteins produced *in situ* using PURE-based TXTL reaction [70]. Alternatively, communication was implemented between emitter TXTL-liposomes and receiver-proteinosomes [71]. By establishing communication between mammalian and bacterial synthetic cell models (synells), Adamala *et al*. provided the first example of diffusive one-way communication between fully synthetic cells of different organisms (Figure 3a) [72^••^]. Such synells comprised liposomes containing either HeLa or *E. coli* extract, as well as quorum sensing DNA circuits. However, to engineer true collective behaviour in populations of synells requires further development of this technology to allow bidirectional exchange of information.

### Natural-artificial cell communication

Schwarz-Schilling *et al*. achieved communication between two distinct droplet populations—encapsulating either TXTL reaction mixture or bacteria [73]. Each population was alternatively equipped with either a receiver circuit or an emitter circuit, so droplets could exchange chemical signals further processed by TXTL or bacteria. Later, Lentini *et al*. implemented communication between lysate-based or PURE-based synthetic cells and various bacteria by combining multiple quorum sensing devices from various species [74] (Figure 3b). In doing so, the authors were able to establish two-way communication between synthetic cells and *Vibrio fischeri* bacteria. Furthermore, authors produced a synthetic cell capable of interfacing communications between various bacterial species which were previously unable to communicate. The synells were programmed to receive a chemical signal from one bacterium (*V. fischeri*), which induced the production of an enzyme disrupting an ongoing communication between an emitter bacterium (*P. aeruginosa*) and a receiver bacterium (engineered *E. coli*), a phenomenon also known as quorum quenching.

## Conclusion and outlook

Cell-free TXTL reactions are becoming an essential tool to prototype novel genetic circuits and can facilitate their integration into living hosts. In combination with microfluidic technologies and the development of novel semipermeable microcapsules, TXTL reactions constitute a unique platform for the study of gene expression and coupling to other cellular processes in a biochemically relevant microenvironment.

It has been established that size and confinement have a considerable impact on TXTL reactions [45,46]. For this reason, more attention should be paid to implementing TXTL reactions in liposomes of defined size and dispersity. In contrast to flow reactors, complex networks have not yet been implemented in liposomes. Possible reasons are the passive diffusion of nutrients, which could be limiting for the TXTL reaction, as well as an absence of active removal of the information. Potential improvements could come from active import and export of substrate and by-products, implementation of a sustained metabolism, all integrated with the enzymatic mRNA and protein degradation methods described in this review. Another remaining challenge is to discover and characterise novel quorum sensors, allowing communication between artificial cells and natural human cells.

Current efforts are exploring possibilities to establish communication between artificial and natural systems, working towards therapeutic applications wherein information exchange between living cells are mediated or altered. We anticipate that micro-compartmentalised TXTL—by accelerating the characterisation of novel genetic circuits and orthogonal molecular communication channels—will have a compelling contribution to the field of synthetic biology.

## Conflict of interest statement

Nothing declared.

## References and recommended reading

Papers of particular interest, published within the period of review, have been highlighted as:

• of special interest
•• of outstanding interest
